# Molecular and Cellular Mechanisms of Osteoporosis

**DOI:** 10.3390/ijms242115772

**Published:** 2023-10-30

**Authors:** Ivan V. Zhivodernikov, Tatiana V. Kirichenko, Yuliya V. Markina, Anton Y. Postnov, Alexander M. Markin

**Affiliations:** Laboratory of Cellular and Molecular Pathology of Cardiovascular System, Petrovsky National Research Centre of Surgery, 119991 Moscow, Russia; kordait-2213@yandex.ru (I.V.Z.); t-gorchakova@mail.ru (T.V.K.); anton-5@mail.ru (A.Y.P.); alexander.markin.34@gmail.com (A.M.M.)

**Keywords:** osteoporosis, inflammation, estrogen deficiency, bone resorption

## Abstract

Osteoporosis is a widespread systemic disease characterized by a decrease in bone mass and an imbalance of the microarchitecture of bone tissue. Experimental and clinical studies devoted to investigating the main pathogenetic mechanisms of osteoporosis revealed the important role of estrogen deficiency, inflammation, oxidative stress, cellular senescence, and epigenetic factors in the development of bone resorption due to osteoclastogenesis, and decreased mineralization of bone tissue and bone formation due to reduced function of osteoblasts caused by apoptosis and age-depended differentiation of osteoblast precursors into adipocytes. The current review was conducted to describe the basic mechanisms of the development of osteoporosis at molecular and cellular levels and to elucidate the most promising therapeutic strategies of pathogenetic therapy of osteoporosis based on articles cited in PubMed up to September 2023.

## 1. Introduction

Osteoporosis is a degenerative systemic disease of the skeleton, characterized by a decrease in bone mass and an imbalance in the microarchitecture of bone tissue, which causes increased bone fragility and, accordingly, osteoporotic fractures [1]. Currently, with an increase in life expectancy, osteoporosis is considered one of the major health problems, leading to unbearable pain, the risk of bone fractures, and even death, which determines the high socio-economic significance of this disease [2]. According to the latest data, more than 200 million people in the world suffer from osteoporosis, and one in three women over the age of 50 years and one in five men have experienced osteoporotic fractures during their lifetime [3]. The main factors leading to the development of osteoporosis include both general factors associated with the natural processes of aging and activation of bone tissue resorption due to deficiency of sex hormones, as well as decreased osteogenesis and microarchitecture disorders due to various external factors, for example, with the administration of glucocorticoids [4]. Experimental and clinical studies devoted to investigating the main pathogenetic mechanisms of osteoporosis development revealed the important role of estrogen deficiency, inflammation, oxidative stress, cellular senescence, and epigenetic factors. The search of articles in PubMed up to September 2023 was conducted to describe the basic mechanisms of the development of osteoporosis at the molecular and cellular levels and to elucidate the most promising therapeutic strategies for the pathogenetic therapy of osteoporosis.

## 2. The Main Pathogenetic Factors of Osteoporosis

### 2.1. Estrogen Deficiency

The mechanism of bone loss associated with estrogen deficiency affects women and men but is more pronounced in women due to a greater decrease in estrogen after menopause. Estrogens are involved in several aspects of bone mass maintenance [5]. Estrogen deficiency leads to an increase in osteoblast apoptosis and inhibition of osteoblast differentiation by different mechanisms. First, estrogens increase the viability and prevent apoptosis of osteoblasts, acting through estrogen receptors, stimulating Wingless integrated type-1 (Wnt)/β-catenin signaling, increasing proliferation and differentiation of osteoblasts. It was shown that inhibition of estrogen receptor-α (ER-α) expression suppresses osteoblast differentiation. Several studies in cell culture and animal models of ER-α-knockout mice demonstrate a critical role of the osteoblast ER-α in bone regeneration and fracture healing [6,7,8].

Moreover, estrogens inhibit the production of several pro-inflammatory cytokines such as tumor necrosis factor-α (TNF-α), interleukins (IL)-1, -4, -6, and IFN-γ, which are involved in the maturation of osteoclasts and play an important role in bone resorption [9]. Estrogens also suppress the production of receptor activator of nuclear factor kappa-Β (NF-κB) ligand (RANKL) and increase the production of osteoprotegerin (OPG) by osteoblasts and lymphocytes, which maintain the RANKL/OPG ratio necessary for balanced bone remodeling [10,11,12].

The important role of IL-17 activated in estrogen deficiency in the development of osteoporosis was demonstrated in numerous studies. IL-17 enhances the secretion of RANKL, TNF-α, and IL-1 by innate immune cells, promoting bone resorption [13]. Postmenopausal women with osteoporosis have higher concentrations of serum IL-17A, RANKL, and OPG and more IL-17-producing CD4+ T-cells in the peripheral blood [14,15]. In an animal model of ovariectomized mice, anti-IL-17 antibody therapy has been shown to prevent bone loss [16,17].

Another study showed that ovariectomy in mice leads to chronic production of the pro-inflammatory cytokines TNF-α and IL-17 by converting memory T-cells into effector cells. Normally, estrogen induces apoptosis of dendritic cells and memory cells. The deficiency of estrogens causes prolonged lifespan of dendritic cells, leading to higher levels of IL-7 and IL-15 and antigen-independent activation of memory T-cells to produce TNFα and IL-17A. Thus, estrogen maintains the homeostasis of memory T-cells and limits their conversion to effector T-cells in the absence of antigens [18]. There is also evidence that estrogen deficiency increases the expression of major histocompatibility complex II (MHC II) molecules on dendritic cells and macrophages, which leads to increased antigen presentation and stimulation of T-cells [19].

**Figure 1:**
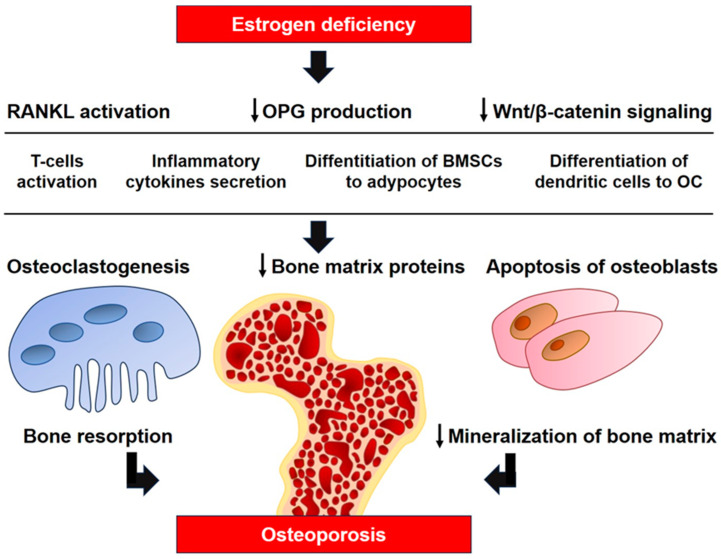
The role of estrogen deficiency in the pathogenesis of osteoporosis. RANKL, receptor activator of nuclear factor kappa-Β ligand; BMSCs, bone marrow mesenchymal stem cells; OC, osteoclast; ↓, decrease.

The major influence of estrogens on T-cell immune factors in the pathogenesis of osteoporosis was demonstrated in a model of athymic mice with T-cell deficiency, which are completely protected from bone loss and increased bone turnover caused by ovariectomy [20,21]. It was demonstrated that estrogens suppress the production of TNF-α by T-cells, preventing osteoclastic bone resorption and bone loss. Ovariectomy leads to increased production of TNF-α by T-cells that stimulate RANKL-induced osteoclastogenesis [20]. Transplantation of T-cells from wild-type mice into T-cell deficient mice makes bone tissue sensitive to ovariectomy, although transplantation of TNF-deficient T-cells does not lead to this result [21]. In addition to the function as an osteoclast activator, RANKL is involved in many components of the immune system in the development of osteoporosis, its interaction with RANK is necessary for the formation of lymph nodes, and it is a key factor stimulating alternative differentiation of osteoclasts from dendritic cells [22,23]. Figure 1 illustrates the major mechanisms of estrogen deficiency in the pathogenesis of osteoporosis.

In the last decade, the mechanism of activation of bone tissue resorption associated with an increase in the level of reactive oxygen species (ROS) during a decrease of estrogen level has been widely discussed [24]. Ovariectomy in animal models leads to an increase in intracellular ROS, while some studies have shown higher serum levels of ROS and decreased levels of endogenous antioxidants in postmenopausal women [25,26,27]. ROS have been observed to stimulate osteoclastogenesis by altering the response of osteoclast precursors to RANKL and inducing additional osteoclastogenic cytokines IL-1, IL-6, and IL-7 [25]. In addition, estrogens modulate signaling pathways involved in redox balance and stimulate the expression of superoxide dismutase (SOD) and glutathione peroxidase (Gpx) [24,28]. The role of oxidative stress in the development of osteoporosis will be discussed below.

A non-immunological component of estrogen-dependent bone loss may be parathyroid cells and C-cells of the thyroid gland, which have receptors for estrogen and respond to its level by changing the secretion of parathyroid hormone and calcitonin according to data of transcriptomic and biochemical analyses [29,30]. Studies of hormonal levels during pregnancy and lactation make it possible to study the dependence of calcitonin and parathyroid hormone on estrogen, but research data are contradictory. During the transition from pregnancy to lactation, the level of estrogen level decreases as well as parathyroid hormone increases, and calcitonin decreases to supply calcium to milk [31,32]. Calcitonin plays a key role in mineral metabolism during lactation and inhibits bone resorption by affecting the osteoclast cytoskeleton. Calcitonin gene-related peptide-alpha (Ctcgrp) null mice lost more minerals during lactation than wild-type mice and administration of exogenous salmon calcitonin reversed this difference [33]. Massive apoptosis of osteoclasts and a significant increase in calcitonin and estrogen levels within 24 h after completion of lactation in rats have been described previously [34,35]. Hormone therapy induced by testosterone propionate and estrogen dipropionate in orchiectomized Wistar rats affected calcitonin-producing thyroid C-cells, increasing C-cell volume and serum calcitonin concentrations. In addition, hormone therapy affected the structure of cancellous bone and reduced the concentration of osteocalcin in urine and serum [29]. Similar results were obtained in the same model using tamoxifen, a selective estrogen receptor modulator, instead of estrogen dipropionate [36]. On the other hand, it is also known that mice with a deletion of the calcitonin gene develop normally and do not demonstrate reduced bone mass, and it is also known that long-term administration of calcitonin forms resistance to it in osteoclasts [37].

### 2.2. Oxidative Stress 

Oxidative stress is one of the essential factors of aging and the etiology of many neurological, cardiovascular, and metabolic diseases, considered as a disturbance of the balance of free radical formation and antioxidant mechanisms. ROS are unavoidable by-products of cellular oxygen metabolism and adenosine triphosphate (ATP) formation, and when excessively accumulated, they cause oxidative stress, which contributes to the development of various diseases [38,39]. It is known that ROS damage cellular and mitochondrial membranes and induce apoptosis. Cells are protected from ROS by recycling them into non-toxic forms using a system of antioxidant enzymes, such as SOD, catalase, and Gpx [38,40]. One of the mechanisms of oxidative stress during aging is a decrease in mitochondrial efficiency, leading to an increased ROS production for ATP synthesis. The important role of mitochondria in aging is associated with their high susceptibility to DNA damage. Nuclear DNA is separated by a double membrane and is better protected from free radical damage. Mitochondrial DNA (mtDNA), located in close proximity to the ROS pool, does not have the protection provided by nucleosomes and DNA repair mechanisms, making mtDNA more susceptible to damage caused by oxidants [41,42]. A direct association of cellular aging, oxidative stress, and mitochondrial damage was demonstrated in some studies. In a recent study, bone marrow mesenchymal stem cells (BMSCs) were exposed to different doses of advanced glycation end products (AGEs) with activation or inhibition of mitophagy, the selective autophagy of mitochondria. The addition of AGEs attenuated the osteogenic potential of BMSCs and supported adipogenesis, while inhibition of mitophagy with cyclosporine A exacerbated this effect. Activation of mitophagy by carbonyl cyanide m-chlorophenylhydrazone resuscitated osteogenesis inhibited by AGEs. BMSCc aging negatively correlated with mitophagy, that was demonstrated by β-galactosidase staining, P53, P21, and P16 protein expression, and immunofluorescence detection of histones H3K9me3 and γ-H2AX. Knockdown of the mitochondrial deacetylase sirtuin 3 by interfering RNA enhanced AGE-induced signs of aging and inhibited mitophagy. Overexpression of sirtuin SIRT3 with an adeno-associated viral vector activated mitophagy and slowed aging. The most important result of the study was the fact that overexpression of SIRT3 in vivo by injection of an adeno-associated viral vector into the tail vein inhibited osteoporosis in SAMP6 mice according to the results of computed tomography, assessment of mineralization, and alkaline phosphatase (ALP) activity [43]. Sirtuins are a family of evolutionarily conserved NAD-dependent proteins with deacetylase or ADP-ribosyltransferase activity and are involved in DNA repair and histone deacetylation. The function of sirtuins is to prevent the aging process, and it was shown to be impaired with age [44]. Sirtuins are often used in studies on the molecular mechanisms of osteoporosis as a correlation sign, an indicator of the effectiveness of different factors in experimental models, and as a promising biochemical target [43,44,45,46,47,48]. In mice, SIRT1 inhibition results in growth retardation and increased spontaneous osteoarthritis with age, which is associated with increased chondrocyte apoptosis [48]. Similar changes were observed in transgenic mice homozygous for the SIRT1-inactivating mutation [47]. SIRT1 in osteoblasts and osteocytes increases trabecular bone mass, while SIRT1 in osteoblast progenitors increases cortical bone by stimulating bone formation at the endocortical surface [44,48].

High levels of ROS negatively affect osteogenesis. Experimental evidence shows that oxidative stress induces apoptosis of osteocytes and osteoblasts, leading to an imbalance in the remodeling process with subsequent altered and insufficient bone formation occurring with aging, glucocorticoid treatment, osteoporosis, and other bone diseases associated with oxidative stress [49,50]. Oxidative stress inhibits osteoblast differentiation through endoplasmic reticulum kinase (ERK)-dependent NF-κB signaling pathways [51]. Osteoblasts can produce antioxidants such as glutathione peroxidase to protect against ROS [52], as well as transforming growth factor β (TGF-β), which is involved in reducing bone resorption [53]. On the other hand, superoxide is generated by osteoclasts for bone resorption, and oxidative stress increases osteoclast differentiation and function [54]. Under the influence of H_2_O_2_, rabbit BMSCs and calvarial osteoblasts showed a decrease in ALP activity, collagen I content, and nuclear phosphorylation of runt-related transcription factor 2 (RUNX2) [55]. There is a clear relationship between the osteogenic differentiation of SaOS-2 cells and the redox status expressed in the ratio of glutathione (GSH) and its oxidized form (GSSG). Increasing the GSH/GSSG ratio stimulated ALP activity, SaOS-2 mineralization, and the expression of RUNX2 and OPG genes [56].

Studies, including the measurements of ROS and AGEs in the blood of people with disorders of the musculoskeletal system, show that their amount correlates with age and the presence of disease. Plasma levels of AGEs, osteoporosis-related biomarkers, and bone mass were assessed in 82 postmenopausal women with osteoporosis or osteopenia, 16 young women with osteopenia, and 43 healthy women without osteoporosis or osteopenia. Higher serum AGEs level was found in patients with osteoporosis or osteopenia compared to healthy women [57]. A larger study included 9203 patients (mean age 57.8 years) and revealed an inverse correlation of AGEs level not only with skeletal mineralization but also with muscle strength of the upper and lower extremities [58].

In this regard, the use of antioxidants in the treatment of osteoporosis looks quite promising, and studies of their effectiveness are being carried out in vivo and in vitro. Melatonin is a powerful endogenous free radical scavenger. The main antioxidant mechanism of melatonin is the scavenging of ROS [59,60]. In addition, melatonin induces the synthesis of antioxidant enzymes, including superoxide dismutase, catalase, glutathione peroxidase, and glutathione reductase [60,61]. Moreover, melatonin promotes osteogenic differentiation of BMSCs through the Wnt/β-catenin pathway and mineralization of the bone matrix [60,62,63]. Melatonin suppresses osteoclastogenesis and induces SIRT1 expression [64,65,66]. It was demonstrated in in vivo experiments that melatonin supplementation in ovariectomized mice had a positive effect on their bone mass [67,68].

Currently, research aimed at studying osteogenesis disorders associated with oxidative stress is a promising direction for the development of therapeutic strategies using various compounds with antioxidant properties, including antioxidants of plant origin [69,70,71,72,73]. New mechanisms of osteoporosis that depend on redox processes are discussed, such as ferroptosis of osteocytes and osteoblasts as a result of iron-dependent lipid peroxidation that occurs in diabetes mellitus. It was shown that fat-soluble vitamin K2 prevents osteocyte death by activating the AMPK/SIRT1 signaling pathway [74].

### 2.3. Cellular Aging

The phenomenon of replicative aging, discovered by Hayflick and Moorehead, extends to pluripotent mesenchymal stem cells of bone tissue, which is reflected in the rate of division, direction of differentiation, and production of the extracellular matrix [75]. The main factor of cellular aging is currently considered to be telomere shortening. In addition to replicative senescence, various toxic effects, primarily interactions with ROS, lead to DNA damage and the immune response to DNA damage [76]. These factors trigger the activation of the p53/p21 Cip1 and p16 Ink4a senescence pathways, which leads to the suppression of cell division [77,78].

It was previously demonstrated that expression of senescence biomarker p16 Ink4a, an inhibitor of cyclin-dependent kinases that plays an important role in cell cycle regulation, and senescence-associated secretory phenotype (SASP) increase with age in osteocytes, and the number of such osteocytes increases with age [78]. During the senescence process, osteogenic differentiation of BMSCs, common progenitors for osteoblasts and adipocytes, is impaired with the shift to adipogenic differentiation. The study aimed to compare the expression of phenotype-specific gene markers in C57BL/6 mice of two age groups, 6–8 months and 20–26 months, showed increased expression of genes associated with adipocyte phenotype and reduced expression of the osteoblast-specific transcription factors, RUNX2 and distal-less homeobox 5 (DLX5), and osteoblast markers, collagen-1 and osteocalcin genes in BMSCs obtained from aged mice. At the same time, U-33/γ2 and U-33/c osteoblasts obtained from BMSCs of 6–8 months old mice accumulated several times more calcium than cells from mice 20–25 months old [79]. Similar results were obtained in the study in BMSCs from Wistar mice aged 21 and 1 month. The mineralization assessed using alizarin red on day 21 of culture as well as ALP activity in BMSCs from 1-month-old rats on days 7 and 10 of cultivation in an osteogenic medium was significantly higher than in BMSCs from rats aged 21 months old. The expression of genes associated with osteogenic differentiation such as RUNX2, osterix, ALP, bone sialoprotein, osteopontin, and osteocalcin, measured on day 10 of cultivation, was also significantly higher in BMSCs from 1-month-old rats [80]. The results of clinical studies comparing the differentiation potential of BMSCs from adipose tissue in patients of four age groups showed an inverse relationship between the osteogenic potential of BMSCs and the age of the study participants. The expression of most osteomarkers, including osteogenic differentiation genes, ALP activity, and mineralization, was higher in the youngest donor group [81,82]. It was also demonstrated in several passages of BMSCs from a 21-year-old donor that the expression of the ALP and collagen-1 genes at passage four was higher than at passage eight, although RUNX2 expression did not differ between passages four and eight. Mineralization was lower in BMSCs of passage eight, indicating the reduced osteogenic potential in aged BMSCs [83]. The study on BMSCs obtained from mice aged 6 days, 6 weeks, and 1 year showed a slight decrease in chondrogenic, adipogenic, and osteogenic potentials at passage six compared to passage one, but the most interesting finding of the study was the dependence of studied parameters on age since the decrease in differentiation potential and chondrogenesis at both passages was more noticeable in the 1-year-aged group [84]. Peroxisome proliferator-activated receptors gene gamma-2 (PPAR-γ2) and other adipogenic genes play multidirectional roles in the differentiation of BMSCs, and it was shown that in aging, the balance shifts toward increased PPAR-γ2 expression and adipogenesis [85].

The use of senolytics or senomorphics can have a beneficial effect on the preservation of bone mass in old age. An increase in bone mass, suppression of bone resorption, and a decrease in the number of cells expressing senescence protein p16Ink4a were reported in 24-month-old INK-ATTAC mice treated with the senolytic drug AP20187 compared to control mice [86]. Quercetin treatment effectively eliminated β-gal staining BMSCs in vitro, which indicated the increased proliferative potential and osteogenic differentiation of senescent BMSCs [87]. Thus, the studies aimed to investigate the age-related changes in bone tissue demonstrate the main pathogenetic role of cellular senescence is a change in the ratio of osteogenic/adipogenic potential of progenitor cells, so the use of senolytics is a promising strategy to ameliorate this factor [88,89].

### 2.4. Epigenetics

Epigenetic studies in the field of osteoporosis research are focused on the investigation of DNA methylation and searching for promising therapeutic targets among modifying proteins [90,91,92]. DNA methyltransferases (DNMTs) can regulate the differentiation of osteoblasts and osteoclasts, playing an important role in the development of osteoporosis [93,94]. During differentiation of BMSCs into osteoblasts, the methylation levels of RUNX2 and SP7 genes decrease, and hypermethylation of the bone morphogenetic protein 2 promoter in osteoblasts can inhibit the expression of genes associated with bone formation [95,96]. In addition, the antagonism of osteogenic and adipogenic differentiation of BMSCs depends on opposite methylation patterns, and their disruption has been proposed as a mechanism for the development of osteoporosis. Transcription regulator histone deacetylase 3 (HDAC3) promotes osteogenesis and inhibits adipogenesis, while histone lysine N-methyltransferase or enhancer of Zeste homolog 2 (EZH2) and histone deacetylase 6 (HDAC6) possess opposite effects [97]. It was shown that BMSCs with retrovirally upregulated EZH2C expression had increased adipogenic potential and decreased RUNX2 transcription compared to control cells. Exposure to interfering RNAs against EZH2C resulted in increased expression of RUNX2. Increased expression of EZH2 led to an increase in tri-methyl histone H3 lysine 27 (H3K27me3) on the RUNX2 promoters, thereby blocking it and suppressing osteogenesis [98,99,100]. In the experiment conducted on 8-week-old ovariectomized C57BL/6 mice, increased expression of EZH2 in BMSCs increased H3K27me3 levels at the Wnt1, Wnt6, and Wnt10A promoters and inhibits Wnt gene transcription, shifting BMSC commitment to adipocytes. Knockdown of EZH2c prevents this shift. In vivo administration of 3-deazaneplanocin A, an H3K27me3 inhibitor, reduces Wnt methylation levels, increases bone production, and suppresses bone marrow fat formation in osteoporotic mice [90,101].

Currently, the search for new enzymes affecting cell differentiation and osteogenesis is a promising direction in research on the pathogenesis of osteoporosis. Methylation of N6-methyladenosine (m6A) regulates the expression of BMSC genes associated with osteogenic differentiation—ALP, RUNX2, SP7, vascular endothelial growth factor, and signaling pathways important in the context of skeletogenesis—PTH/Pth1r, PI3K-Akt, and Wnt/β-Catenin [102]. Knockout of methyltransferase-like 3 (Mettl3) leads to a decrease in bone mass in mice, a decrease in osteogenic potential and increased adipogenic differentiation of BMSCs, and accumulation of adipose tissue in the bone marrow, while overexpression of Mettl3 protects mice from osteoporosis caused by estrogen deficiency [103]. It has also been reported that the methylation inhibitor 5-AZA-2-deoxycytidine (5-Aza-dC) is effective against DNMT1, activation of the expression of DLX5, osterix, ALP, osteocalcin genes, and osteogenic differentiation of BMSCs [93,97,104]. Methylation analysis of 44 patients using next-generation sequencing identified five proteins significant in osteoporosis: Zinc Finger Protein 267 (ZNF267), Actin Binding LIM Protein Family Member 2 (ABLIM2), Ras Homolog Family Member J (RHOJ), Cyclin-Dependent Kinase-Like 5 (CDKL5), and Programmed Cell Death 1 (PDCD1) [105,106]. A study on trabecular bone samples from 12 patients using immunoprecipitation of methylated DNA revealed four genes differentially expressed in osteoporosis—PPIL3, NIF3L1, SMTN, and CALHM2 [107].

Epigenetic mechanisms are critical regulators of BMSC differentiation that deteriorate during aging, so therapeutic or regenerative strategies based on epigenetic regulation of BMSC aging are considered promising for maintaining BMSC homeostasis in elderly people or patients with bone diseases [97].

### 2.5. Genetic Factors

The physiological characteristics leading to the osteoporotic phenotype are inherited through multiple alleles, and a family history of osteoporotic fractures is generally considered a significant risk factor for osteoporosis. According to various estimates, the impact of hereditary factors in the development of osteoporotic fractures reaches 85% [108]. Despite its high heritability, osteoporosis is not considered a hereditary pathology since it is influenced by various metabolic, alimentary, and other factors, including vitamin D levels. Some of these factors are also under genetic control, in particular, genes affecting BMD. Osteoporosis is rarely caused by single gene mutations, which can negatively affect bone mineralization and osteoblast differentiation and function [109,110]. In addition, rarer cases of monogenic osteoporosis are associated with mutations in genes involved in WNT1 signaling. Thus, biallelic nonsense or frameshift mutations of WNT1 can also lead to osteogenesis imperfecta, earlier onset of fragility fractures, and the development of early-onset osteoporosis [111]. The most common causes of osteoporosis in the general population are multiple mutations representing a complex genetic architecture that is still not fully understood. Genome-wide association studies (GWAS) in bone have identified hundreds of loci that are associated with BMD. The most common monogenic mutations are type I collagen abnormalities. Thus, inherited autosomal dominant mutations and de novo mutations of the COL1A1 and COL1A2 genes are the causes of osteogenesis imperfecta in 85–90% of cases of osteoporosis and osteoporotic fractures [112]. Research suggests that various mutations in genes CLCN7, GALNT3, IBSP, LTBP3, RSPO3, and SOX4 cause decreased BMD [113]. TNFRSF11B, LRP5, RUNX2, SP7, SOST, DKKI, and ESR1 have a strong influence on osteoporosis development, according to GWAS data. Among the 19 human WNT ligands, WNT16 affects cortical bone properties and homeostasis, and patients with early-onset osteoporosis have been observed to have a high frequency of heterozygous mutations in the WNT1 gene [114]. The sclerostin SOST gene, an inhibitor of the Wnt/β-catenin signaling pathway, which is essential for the maintenance of bone mass, is well known for multiple single nucleotide polymorphisms causing variability in bone mineral density [115]. The role of a combination of genetic variants of the regulatory proteins OPG and LRP5, which also contribute to a decrease in BMD, has been shown [116]. Several studies demonstrate the contribution of low-frequency and rare genetic variants to decreased bone mineral density in the general population. For example, a low-frequency noncoding variant near the novel EN1 locus has a stronger effect on BMD and causes the development of lumbar spine fractures compared with other genetic variants [117]. Bone metabolic disorders can be caused by variations in the nuclear and mitochondrial DNA copy number [118]. Thus, the combination of different genetic variants involving multiple loci associated with bone mineral density and fracture risk may have a direct impact on the development of osteoporosis in the general population.

Searching for correlations in gene expression profiling databases reveals an association between osteoporosis and Parkinson’s disease. The genes SNAP25, AQP4, SV2B, KCND3, and ABCA2 have important diagnostic value for both diseases [119]. Common genes associated with osteoporosis and chronic gastritis were identified: CD163, CD14, CCR1, CYBB, CXCL10, SIGLEC1, LILRB2, IGSF6, MS4A6A, and CCL8. The correlation between osteoporosis and chronic gastritis may be due to the ability of parathyroid hormone to stimulate gastrin secretion, as well as an increased pro-inflammatory profile in patients with chronic gastritis, which stimulates bone resorption [120]. The association of osteoporosis with chronic colitis and Crohn’s disease was shown in a meta-analysis of 30 GWAS. The incidence of fractures in people with inflammatory bowel disease is 40% higher than in the general population [121].

### 2.6. Inflammation

**Figure 2:**
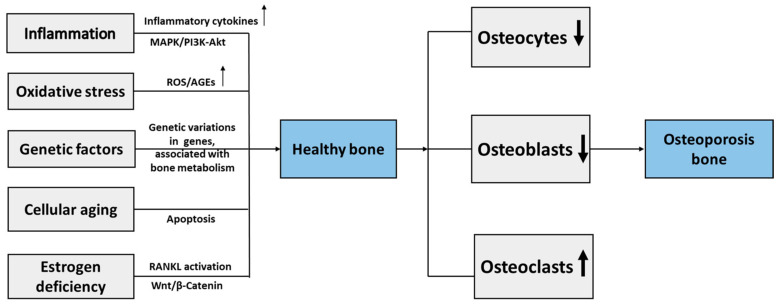
Molecular and cellular mechanisms of osteoporosis. RANKL, receptor activator of nuclear factor kappa-Β ligand; ROS, reactive oxygen species; AGEs, advanced glycation end products; MAPK, mitogen-associated protein kinase; PI3K-Akt, phosphoinositide 3-kinase/protein kinase B; ↓, decrease; ↑, increase.

Recent findings in osteoimmunology demonstrate a key role of inflammatory factors in described mechanisms of osteoporosis. The chronic inflammatory conditions induced by aging and estrogen deficiency activate the NLRP3 inflammasome, which leads to inflammatory mediators oversecretion and stimulates the inflammatory response. NLRP3 inflammasome plays an important role in the development of osteoporosis by reducing differentiation, causing dysfunction of the osteoblasts, and accelerating osteoclasts, thus promoting bone resorption and impaired bone formation [122]. The study in postmenopausal women with osteoporosis demonstrated that single-nucleotide variants in NLRP3 inflammasome pathway genes activating pro-inflammatory cytokines IL-1β and IL-18 production are associated with osteoporosis severity [123]. Increased secretion of inflammatory cytokines, in particular, TNF-α and IL-1β, induces MAPK activation that leads to reduced osteoblastogenesis, resulting in defective bone remodeling [124]. Several studies on treatment approaches aimed to modulate NLRP3 inflammasome activity and inflammatory cytokines production reported beneficial effects of therapy on bone metabolism and osteoporosis development [125,126]. Figure 2 demonstrates the main cellular and molecular mechanisms of osteoporosis.

In fact, estrogen deficiency is a crucial factor in triggering the cascade of inflammatory mechanisms in the pathogenesis of osteoporosis, as described in the first section of the review. Estrogen deficiency causes the activation of RANKL signaling, resulting in increased production of inflammatory cytokines by activation of immune cells, including T-lymphocytes, that leads to osteoclastogenesis [5]. In this regard, the research of the RANKL/RANK/OPG system is a key scope of osteoimmunology [127,128].

The important role of inflammatory factors underlies the functioning of the gut microbiota—bone axis involved in bone metabolism by affecting anti-inflammatory pathways by releasing various metabolites, in particular, short-chain fatty acids (SCFA) [129]. The effectiveness of SCFA therapy in terms of prevention of inflammation-induced bone resorption was confirmed in several studies on cellular and animal models [130].

## 3. Modern Therapeutic Approaches for the Treatment of Osteoporosis

Increasing advances in the research of osteoporosis allowed the development of various therapeutic strategies for pathogenetic prevention and treatment of osteoporosis. Table 1 presents the most widely used approaches as well as promising directions in osteoporosis therapy.

### 3.1. Bisphosphonates

Currently, the first-line preparations for the treatment of osteoporosis are antiresorptive drugs, in particular bisphosphonates, which prevent bone resorption by osteoclasts, thereby increasing bone density and reducing the risk of fractures [148]. Widely used preparations of this group for the prevention and treatment of postmenopausal osteoporosis are preparations of the second and third-generation approved their effectiveness in clinical studies alendronate, ibandronate, risedronate, and zoledronic acid [149]. The mechanism of action of these preparations is based on the binding and inhibition of the activity of farnesyl pyrophosphate synthase (FPP), a key enzyme in the mevalonic acid pathway, which is critical for the production of cholesterol, other sterols, and isoprenoid lipids. The inhibition of FPP leads to the suppression of post-translational modification of proteins that play a central role in maintaining the cytoskeleton and regulation of the osteoclast activity that causes osteoclast apoptosis [150,151]. Thus, bisphosphonates are able to selectively inhibit osteoclast activity and thereby slow down bone resorption, especially in postmenopausal osteoporosis [152].

### 3.2. RANKL Inhibitors

The main preparation of the group of RANKL inhibitors approved and widely used for the treatment of postmenopausal osteoporosis is denosumab, a monoclonal antibody that binds to RANKL and prevents its binding to the RANK receptor [135]. Since RANKL is a major mediator of bone resorption that initiates a signaling cascade essential for differentiation, activation, and survival of osteoclasts. Denosumab suppresses osteoclast function by inhibiting osteoclastogenesis and the return of nonresorbable osteomorphs to osteoclasts. However, it was shown that high doses of RANKL inhibitors can transform accumulating osteomorphs into active osteoclasts that lead to bone resorption, which is accompanied by fractures [136]. The effectiveness of denosumab has been studied in comparison with bisphosphonates and found that it was associated with a significantly greater reduction in the risk of vertebral and other fractures than alendronate or ibandronate, but no differences were found compared with zoledronate [153]. Clinical studies have shown that long-term therapy with denosumab for up to 10 years was associated with an increase in bone mineral density (BMD), a low incidence of fractures, and a low level of side effects [154]. In addition to denosumab, other compounds that affect RANKL are being identified and developed. However, these drugs are only at the development stage. For example, EPZ compounds, inhibitors of protein arginine methyltransferase 5, affect RANKL-induced osteoclast differentiation through molecular mechanisms in in vitro experiments. Namely, EPZ compounds reduce the nuclear translocation of NF-κB by inhibiting the dimethylation of the p65 subunit, which ultimately prevents osteoclast differentiation and bone resorption [155]. Moreover, it was found in in vitro studies that the XL019 compound, a Janus kinase inhibitor, inhibits RANKL-induced osteoclastogenesis by suppressing mitogen-activated protein kinase signaling and reducing the expression of osteoclast-specific genes and proteins, thereby preventing bone resorption [156]. Other studies are devoted to the investigation of inhibitors of RANKL through LGR4 signaling and the generation of anti-RANKL antibodies, which may be a promising target for the regulation of osteoclast resorption [157].

### 3.3. Parathyroid Hormone Analogs

The main preparations in the group of parathyroid hormones (PTH) analogs include teriparatide, an analog of PTH, and abaloparatide, an analog of the PTH-related peptide. The main mechanism of action of PTH analogs is the binding of the PTH receptor (PTH1R), which affects the G protein/cAMP signaling pathway and activates protein kinase A [138]. In addition, when teriparatide and abaloparatide bind to PTH1R, several signaling pathways, Gq/phospholipase C/Ca^2+^ and β-arrestin/ERK, are activated, leading to the activation of transcription factors of genes underlying the anabolic response [158]. PTH analogs enhance the formation of osteoblasts, thereby increasing bone density, improving bone strength, and reducing the risk of fractures [139]. However, despite the high effectiveness of PTH analogs, discontinuation of therapy leads to a reduction of bone mineral density and requires further antiresorptive therapy to enhance or maintain the achieved effects [140]. Clinical studies of teriparatide in osteoporosis showed that despite the early positive effect on bone formation, the “anabolic window” period and subsequent bone resorption subsequently occurred [159]. It has been revealed that receptor activity-modifying proteins (RAMPs) can bind to G protein-coupled receptors (GPCRs), including the PTH1R receptor, thereby affecting bone metabolism that allows to consider this mechanism as a promising target for specific modulation of anabolic processes in bone tissue [160].

### 3.4. Antisclerostin Antibodies 

Preparations of the group antisclerostin antibodies affect the protein sclerostin, produced and secreted by mature osteocytes, which blocks the activation of the osteogenic Wnt-signaling pathway, thereby controlling bone formation by osteoblasts. Sclerostin is a bone morphogenetic protein (BMP) antagonist encoded by the SOST gene that binds to the Wnt coreceptors LRP 5/6 [161]. As described above, inhibition of the canonical Wnt-signaling pathway in mature osteoblasts/osteocytes reduces the levels of OPG, the decoy receptor for RANKL, leading to increased osteoclast differentiation and bone resorption [162]. Antisclerostin antibodies slow down the binding of sclerostin to LRP 5/6, increasing the concentration of β-catenin and reducing the negative suppression of Wnt-induced responses [141]. The most well-known sclerostin inhibitor, approved by the FDA and approved in clinical trials, is romosozumab. This preparation increases the number of osteoblasts, improves mechanical strength by increasing bone mass, improves structural and architectural characteristics, and optimizes the composition of bone tissue [163,164]. However, romosozumab has a limited period of effectiveness, which requires concomitant antiresorptive therapy [164]. It was shown that sequential use of romosozumab followed by denosumab may be a promising regimen for the treatment of osteoporosis [165]. It is important that romosozumab administration increases the incidence of adverse cardiovascular events, and therefore, its use should be avoided in patients at high risk of cardiovascular or cerebrovascular diseases [142]. In addition to romosozumab, two other antibodies to sclerostin, setrusumab and blosozumab, have been studied in clinical trials but are not currently used in clinical practice [166,167].

### 3.5. Selective Estrogen Receptor Modulators (SERM) 

The most studied preparations of the group of selective estrogen receptor modulators (SERMs) are raloxifene and bazedoxifene. These preparations bind to estrogen receptors and act as receptor antagonists in the uterus and mammary gland, and at the same time as agonists, regulating bone and lipid metabolism [168]. The beneficial effects of raloxifene in osteoporosis treatment are an increase in bone mass and mineral density due to a decrease in bone resorption [143]. Moreover, raloxifene possesses additional positive effects on the cardiovascular system, including the reduction of serum levels of LDL cholesterol, fibrinogen, and lipoprotein A, and increases the level of HDL cholesterol. However, the main side effects of raloxifene, such as hot flashes and venous thromboembolism, limit its use in some patients [169,170]. However, SERM preparations are successfully used in postmenopausal osteoporosis [145].

### 3.6. Calcitonin

Calcitonin preparations are derivatives of human calcitonin, also used to treat postmenopausal osteoporosis. Calcitonin, a peptide hormone of the thyroid gland, has the ability to inhibit the activity of osteoclasts, thereby reducing the loss of bone mineral density and helping to reduce the risk of osteoporotic fractures [144]. Currently, due to low efficiency and side effects, calcitonin is rarely used in clinical practice but can be used for the treatment of acute osteoporotic fractures [171].

### 3.7. Promising Directions of Osteoporosis-Targeting Therapy

Recently, the cGAS-STING signaling pathway has attracted attention as an important factor in the cellular inflammatory response and a new target of anti-inflammatory therapy. Stimulator of interferon genes (STING) triggers innate immune activation by upregulation of NF-κB transcription and producing type I interferon independently of Toll-like receptors along with downregulation of IFN-β production. Moreover, activation of STING induces the transcription of inflammatory genes and increases the secretion of pro-inflammatory cytokines, thereby triggering chronic inflammation and promoting the development of inflammatory and autoimmune diseases [172]. It was shown that targeting STING/NF-κB in osteoporosis treatment is a promising therapeutic approach to reduce bone resorption by inhibiting osteoclast differentiation [146].

Semaphorins are extracellular signaling proteins targeting plexin receptors. Semaphorins are key regulators of the morphology and mobility of cells in the nervous, cardiovascular, immune, endocrine, musculoskeletal, and other systems [173]. The molecule semaphorin 4D (Sema4D) produced by osteoclasts suppresses bone formation through the Plexin-B1/IGF-1 signaling pathway and also promotes osteoclast resorption and osteoclastogenesis by binding CD72 to osteoclast precursors [174,175]. In contrast, Sema3A produced by osteocytes and osteoblasts can inhibit Plcγ2 and M-CSF-induced osteoclast differentiation and stimulate Plexin-A and neuropilin 1 (Nrp1) through canonical Wnt/β-catenin signaling, thus acting as a potent osteogenic and osteoprotective factor [176]. It was demonstrated in animal experiments that the administration of Sema4D-specific siRNA and Sema3A-specific agonists enhanced bone formation and reduced bone resorption in healthy and ovariectomized mice [147]. The revealed dual regulatory effects of semaphorins on bone remodeling attract special attention, which may indicate great prospects for the development of drugs based on them for the treatment of osteoporosis.

Pyrroloquinoline quinone (PQQ), which is considered a catalyst for redox reactions and can scavenge ROS and ameliorate oxidative stress, shows high efficiency in maintaining osteogenesis in murine models. In the model of ovariectomized C57BL/6 mice administered subcutaneous estradiol or supplemented with PQQ, there was no difference in parameters of bone mass, mineralization, osteoresorption, biomechanics, redox balance, and cellular aging between mice with PQQ in the diet and mice who received estrogens. These results confirm the fact that estrogen deficiency reduces antioxidant defense and induces oxidative stress [177].

In another study in mice recessively homozygous for B cell-specific Moloney murine leukemia virus integration site 1 (BMI-1), which normally exhibit premature aging and osteoporosis, the addition of PQQ had a positive effect on bone mass, osteonectin, and collagen 1 content, decreased levels of ROS, and ALP activity. In addition, Western blot analysis of P16, P19, P21, P27, P53, γH2AX, and caspase-3 proteins confirmed reduced bone resorption, DNA damage, and apoptosis [178]. Similar results were obtained in testosterone-deficient mice, demonstrating that the addition of PQQ in the diet compensated the effect of orchiectomy on osteoblastogenesis and the expression of proteins associated with DNA repair [179].

Calcium-based nanomaterials used not only as locally applied approaches, such as implant coatings and filling materials for osteoporotic bone regeneration, but also as preparations for antiosteoporosis treatment are perspective treatment strategy for delaying osteoporosis progression due to their pro-osteogenic properties [180].

Many studies indicate the role of long noncoding RNAs (lncRNAs) in signaling processes affecting bone formation. lncRNAs are involved in transcription, translation, regulation of gene expression, cell differentiation, and cell cycle regulation. According to recent studies, lncRNAs promote osteogenesis through signaling pathways associated with osteogenesis transcription factors and microRNAs. Exosomes and binding to bone implants are being considered promising approaches for lncRNAs implementation [181].

HMGA1 encodes a non-histone regulatory region of chromatin, which is involved in its organization and gene transcription. In vivo and in vitro experiments showed that HMGA1 expression increases during osteogenesis of rat BMSCs and decreases during ovariectomy. Bone loss during ovariectomy in rats was compensated by the introduction of a lentivirus inserting HMGA1 into the bone marrow cavity; therefore, HMGA1 is considered a potential gene therapy target for the treatment of osteoporosis [182]. The clinical potential of the adeno-associated virus, an inhibitor of the WNT/β-catenin signaling pathway antagonists SHN3 and SOST, was demonstrated in terms of enhanced osteoblast function and bone formation [183].

The potential of stem cells in osteoporosis therapy is aimed primarily at stimulating bone formation due to their proliferative and anti-inflammatory properties. Stem cells differentiate into osteoblasts. In animal models, stem cell injection can improve bone microstructure and bone density, increase ALP activity, activate OPG production, and inhibit TNF-α and RANKL expression. Currently, stem cells are at the stage of animal experiments, and ethical controversies are raised in terms of this type of therapy [184]. The metabolites of stem cells, such as the secretome and conditioned medium, are increasingly being considered since the intervention of BMSCs may still be associated with the risk of immune complications. In this regard, the use of amniotic mesenchymal stem cell products (AMSCs), which contain cytokines and growth factors, namely bFGF, VEGF, TGF-β, EGF, KGF, HGF, FGF7, and BMP-2, playing an important role in the healing of bone defects is investigated [185]. Another promising option for using the BMSC secretome is exosomes, secretory vesicles sized 30–120 nm, which contain proteins, lipids, and nucleic acids and serve as important mediators of intercellular communication. Exosomes obtained from BMSCs have been shown to increase the expression of osteodifferentiation genes, delivering growth factors and microRNAs that possess a positive effect on bone regeneration [186].

### 3.8. Personalized Approaches in Osteoporosis Treatment

Because there are many different mechanisms leading to the osteoporotic phenotype, there are a variety of preparations targeting these mechanisms, but the effectiveness of combinations of these preparations varies from one patient to another despite having similar effects. The number of preparations can be prescribed only for postmenopausal osteoporosis and are not applicable to other groups of patients. Widely used bone resorption inhibitors, bisphosphonates, may not provide a therapeutic effect during the first 2 years of use in 20–40% of all patients. Patient genetic profiles are currently being studied in an attempt to generate predictive patterns for response to therapy and to personalize the treatment [187,188]. In therapy aimed at reducing bone resorption, the adjuvant use of antioxidants has been proposed as an alternative to the use of antiresorptive drugs to inhibit osteoclast activity, which could restore the balance between osteoclasts and osteoblasts and the process of physiological remodeling [189].

The development of osteoporosis is strongly associated with individual risk factors, including nutrition, body weight, lifestyle factors such as smoking and inactivity, geographic factors associated with insufficient sunlight exposure, and others [190,191]. In this regard, dietary modifications, exercise recommendations, and lifestyle changes should be included in the personalized treatment strategies for osteoporosis. The results of the study of lifestyle modification intervention along with pharmacotherapy showed the beneficial effect of lifestyle modification on BMD in postmenopausal osteoporotic women [192]. The evaluation of vitamin D status is quite relevant for timely diagnostics of vitamin D deficiency in osteoporosis prevention [193]. Whole-body vibration therapy is considered a promising physiotherapeutic measure to increase bone mass and density in age-related osteoporosis since it is effectively used in space medicine [194]. Among various risk factors of osteoporosis, gut microbiome disturbance affects osteoporosis development due to immune and endocrine mechanisms as well as via disruption of the gut–bone axis signaling [195]. So, the personalized use of pre- and probiotics and other therapies to restore the gut microbiome is highly recommended in therapeutic interventions in osteoporosis. In particular, several studies demonstrated the beneficial effect of fecal microbiota transplantation from healthy donors to recipients with gut microbiota imbalance on osteoporosis development in experimental models and clinical trials [196].

## 4. Conclusions

Thus, the aging of the organism is a stimulating factor for processes associated with the development of osteoporosis. Decreased production of sex hormones, as one of the aspects of aging, induces bone resorption through increased production of pro-inflammatory mediators. An increase in the level of ROS with age prevents osteogenic differentiation and promotes the increase of osteoclasts; at the same time, the efficiency of osteogenic differentiation of progenitor cells decreases during aging with a shift to adipogenesis. At an earlier age, factors associated with estrogen deficiency and inflammation are of key importance in the pathogenesis of osteoporosis; at a later age, the reduction of osteoblast differentiation becomes the main factor in the pathogenesis of osteoporosis. These mechanisms also depend on the individual hormonal status of the organism, so the prospects for the treatment of osteoporosis are believed to be the further development of personalized medicine. Currently, there is a wide range of preparations that affect various cellular and molecular mechanisms of osteoporosis; however, treatment with existing preparations is associated with the risk of serious side effects, so further research aimed at the development of new pathogenetic approaches to the treatment of osteoporosis is extremely relevant.

## Figures and Tables

**Table 1:** Characteristics of the main preparations for osteoporosis therapy.

Drug	Mechanism of Action/Effects	Comments
Bisphosphonates	Selectively inhibit the enzyme farnesyl pyrophosphate synthase in osteoclasts, leading to their apoptosis [131]	Gastrointestinal adverse effects [132] Atypical fractures [133] Not recommended for patients with low creatinine clearance [134]
RANKL inhibitors	Inhibits the binding of RANKL to its receptor, inhibits osteoclastogenesis [135,136]	Duration of denosumab administration increases the rate of bone resorption after treatment cessation [137]
PTH analogues	Binds the PTH receptor (PTH1R), activates protein kinase A, enhances the formation of osteoblasts [138,139]	Decreases bone mineral density after withdrawal [140]
Antisclerostin antibodies	Slow down the binding of sclerostin to LRP5/6, reduce blocking of the osteogenic Wnt pathway, increases the number of osteoblasts [141]	Increase the risk of cardiovascular events [142]
Hormone therapy	Binds to estrogen receptors [143]	Used for postmenopausal osteoporosis [143]
Calcitonin	Inhibit osteoclast activity [144]	Used for postmenopausal osteoporosis, low efficiency [144,145]
Targeting STING	Activation of STING/IFN-β [146]	It is a potential target
Targeting semaphorins	Modulate plexin receptors, enhanced bone formation, and reduced bone resorption [147]	It is a potential target

## References

[1] Yang J., Jiang T., Xu G., Liu W. (2023). Bibliometrics Analysis and Visualization of Sarcopenia Associated with Osteoporosis from 2000 to 2022. J. Pain Res..

[2] Wang H., Luo Y., Wang H., Li F., Yu F., Ye L. (2023). Mechanistic Advances in Osteoporosis and Anti-Osteoporosis Therapies. MedComm.

[3] Sözen T., Özışık L., Başaran N.Ç. (2017). An Overview and Management of Osteoporosis. Eur. J. Rheumatol..

[4] Liu J., Xu D., Liu L., Huang C., Guo Z., Zhang D., Wei L. (2023). Regular Sling Core Stabilization Training Improves Bone Density Based on Calcium and Vitamin D Supplementation. BMC Musculoskelet. Disord..

[5] Fischer V., Haffner-Luntzer M. (2022). Interaction between Bone and Immune Cells: Implications for Postmenopausal Osteoporosis. Semin. Cell Dev. Biol..

[6] Lin Y., Xiao L., Zhang Y., Li P., Wu Y., Lin Y. (2019). MiR-26b-3p Regulates Osteoblast Differentiation via Targeting Estrogen Receptor α. Genomics.

[7] Steppe L., Krüger B.T., Tschaffon M.E.A., Fischer V., Tuckermann J., Ignatius A., Haffner-Luntzer M. (2021). Estrogen Receptor α Signaling in Osteoblasts Is Required for Mechanotransduction in Bone Fracture Healing. Front. Bioeng. Biotechnol..

[8] Ikedo A., Imai Y. (2021). Estrogen Receptor α in Mature Osteoblasts Regulates the Late Stage of Bone Regeneration. Biochem. Biophys. Res. Commun..

[9] Vrachnis N., Zygouris D., Vrachnis D., Antonakopoulos N., Fotiou A., Panagopoulos P., Kolialexi A., Pappa K., Mastorakos G., Iliodromiti Z. (2021). Effects of Hormone Therapy and Flavonoids Capable on Reversal of Menopausal Immune Senescence. Nutrients.

[10] Cheng C.H., Chen L.R., Chen K.H. (2022). Osteoporosis Due to Hormone Imbalance: An Overview of the Effects of Estrogen Deficiency and Glucocorticoid Overuse on Bone Turnover. Int. J. Mol. Sci..

[11] Wang L.T., Chen L.R., Chen K.H. (2023). Hormone-Related and Drug-Induced Osteoporosis: A Cellular and Molecular Overview. Int. J. Mol. Sci..

[12] Tian H., Jiang T., Yang K., Ning R., Wang T., Zhou Q., Qian N., Huang P., Guo L., Jiang M. (2022). α-Asarone Attenuates Osteoclastogenesis and Prevents Against Oestrogen-Deficiency Induced Osteoporosis. Front. Pharmacol..

[13] Zhang J., Fu Q., Ren Z., Wang Y., Wang C., Shen T., Wang G., Wu L. (2015). Changes of Serum Cytokines-Related Th1/Th2/Th17 Concentration in Patients with Postmenopausal Osteoporosis. Gynecol. Endocrinol..

[14] Molnár I., Bohaty I., Somogyiné-Vári É. (2014). IL-17A-Mediated SRANK Ligand Elevation Involved in Postmenopausal Osteoporosis. Osteoporos. Int..

[15] Zhao R., Wang X., Feng F. (2016). Upregulated Cellular Expression of IL-17 by CD4+ T-Cells in Osteoporotic Postmenopausal Women. Ann. Nutr. Metab..

[16] Shukla P., Mansoori M.N., Singh D. (2018). Efficacy of Anti-IL-23 Monotherapy versus Combination Therapy with Anti-IL-17 in Estrogen Deficiency Induced Bone Loss Conditions. Bone.

[17] Mansoori M.N., Shukla P., Singh D. (2017). Combination of PTH (1-34) with Anti-IL17 Prevents Bone Loss by Inhibiting IL-17/N-Cadherin Mediated Disruption of PTHR1/LRP-6 Interaction. Bone.

[18] Cline-Smith A., Axelbaum A., Shashkova E., Chakraborty M., Sanford J., Panesar P., Peterson M., Cox L., Baldan A., Veis D. (2020). Ovariectomy Activates Chronic Low-Grade Inflammation Mediated by Memory T Cells, Which Promotes Osteoporosis in Mice. J. Bone Miner. Res..

[19] Ma’Arif B., Mirza D.M., Hasanah M., Laswati H., Agil M. (2020). Antineuroinflammation Activity of N-Butanol Fraction of Marsilea Crenata Presl. in Microglia HMC3 Cell Line. J. Basic Clin. Physiol. Pharmacol..

[20] Cenci S., Weitzmann M.N., Roggia C., Namba N., Novack D., Woodring J., Pacifici R. (2000). Estrogen Deficiency Induces Bone Loss by Enhancing T-Cell Production of TNF-Alpha. J. Clin. Investig..

[21] Roggia C., Gao Y., Cenci S., Weitzmann M.N., Toraldo G., Isaia G., Pacifici R. (2001). Up-Regulation of TNF-Producing T Cells in the Bone Marrow: A Key Mechanism by Which Estrogen Deficiency Induces Bone Loss in Vivo. Proc. Natl. Acad. Sci. USA.

[22] Monteiro A.C., Bonomo A. (2021). Dendritic Cells Development into Osteoclast-Type APCs by 4T1 Breast Tumor T Cells Milieu Boost Bone Consumption. Bone.

[23] Yang X., Wang X., Chi M., Zhang M., Shan H., Zhang Q.H., Zhang J., Shi J., Zhang J.Z., Wu R.M. (2019). Osteoprotegerin Mediate RANK/RANKL Signaling Inhibition Eases Asthma Inflammatory Reaction by Affecting the Survival and Function of Dendritic Cells. Allergol. Immunopathol..

[24] Bonaccorsi G., Piva I., Greco P., Cervellati C. (2018). Oxidative Stress as a Possible Pathogenic Cofactor of Post-Menopausal Osteoporosis: Existing Evidence in Support of the Axis Oestrogen Deficiency-Redox Imbalance-Bone Loss. Indian J. Med. Res..

[25] Cervellati C., Bergamini C.M. (2016). Oxidative Damage and the Pathogenesis of Menopause Related Disturbances and Diseases. Clin. Chem. Lab. Med..

[26] Serrano Mujica L.K., Stein C.S., Miyazato L.G., Valente F., Premaor M.O., Antoniazzi A.Q., Moresco R.N., Comim F.V. (2021). Ovariectomy Improves Metabolic and Oxidative Stress Marker Disruption in Androgenized Rats: Possible Approach to Postmenopausal Polycystic Ovary Syndrome. Metab. Syndr. Relat. Disord..

[27] Chandankhede M., Gupta M., Pakhmode S. (2021). Assessment of Psychological Status and Oxidative Stress in Postmenopausal Women: A Cross-Sectional Study. J. Menopausal. Med..

[28] Zhang C., Li H., Li J., Hu J., Yang K., Tao L. (2023). Oxidative Stress: A Common Pathological State in a High-Risk Population for Osteoporosis. Biomed. Pharmacother..

[29] Filipović B., Šošić-Jurjević B., Ajdžanović V., Pantelić J., Nestorović N., Milošević V., Sekulić M. (2013). The Effects of Sex Steroids on Thyroid C Cells and Trabecular Bone Structure in the Rat Model of Male Osteoporosis. J. Anat..

[30] Haglund F., Ma R., Huss M., Sulaiman L., Lu M., Nilsson I.L., Höög A., Juhlin C.C., Hartman J., Larsson C. (2012). Evidence of a Functional Estrogen Receptor in Parathyroid Adenomas. J. Clin. Endocrinol. Metab..

[31] Scioscia M.F., Zanchetta M.B. (2023). Recent Insights into Pregnancy and Lactation-Associated Osteoporosis (PLO). Int. J. Womens Health.

[32] Carsote M., Turturea M.R., Valea A., Buescu C., Nistor C., Turturea I.F. (2023). Bridging the Gap: Pregnancy-And Lactation-Associated Osteoporosis. Diagnostics.

[33] Woodrow J.P., Sharpe C.J., Fudge N.J., Hoff A.O., Gagel R.F., Kovacs C.S. (2006). Calcitonin Plays a Critical Role in Regulating Skeletal Mineral Metabolism during Lactation. Endocrinology.

[34] Miller S.C., Bowman B.M. (2007). Rapid Inactivation and Apoptosis of Osteoclasts in the Maternal Skeleton during the Bone Remodeling Reversal at the End of Lactation. Anat. Rec..

[35] Sondergaard B.C., Oestergaard S., Christiansen C., Tankó L.B., Karsdal M.A. (2007). The Effect of Oral Calcitonin on Cartilage Turnover and Surface Erosion in an Ovariectomized Rat Model. Arthritis Rheum..

[36] Filipović B., Šošić-Jurjević B., Ajdžanović V., Živanović J., Isenović E., Popovska-Perčinić F., Milošević V. (2015). Tamoxifen Stimulates Calcitonin-Producing Thyroid C-Cells and Prevents Trabecular Bone Loss in a Rat Model of Androgen Deficiency. J. Anat..

[37] Hoff A.O., Catala-Lehnen P., Thomas P.M., Priemel M., Rueger J.M., Nasonkin I., Bradley A., Hughes M.R., Ordonez N., Cote G.J. (2002). Increased Bone Mass Is an Unexpected Phenotype Associated with Deletion of the Calcitonin Gene. J. Clin. Investig..

[38] Chaudhary P., Janmeda P., Docea A.O., Yeskaliyeva B., Abdull Razis A.F., Modu B., Calina D., Sharifi-Rad J. (2023). Oxidative Stress, Free Radicals and Antioxidants: Potential Crosstalk in the Pathophysiology of Human Diseases. Front. Chem..

[39] Zaric B.L., Macvanin M.T., Isenovic E.R. (2023). Free Radicals: Relationship to Human Diseases and Potential Therapeutic Applications. Int. J. Biochem. Cell Biol..

[40] Jing J., Peng Y., Fan W., Han S., Peng Q., Xue C., Qin X., Liu Y., Ding Z. (2023). Obesity-Induced Oxidative Stress and Mitochondrial Dysfunction Negatively Affect Sperm Quality. FEBS Open Bio.

[41] Vatner S.F., Zhang J., Oydanich M., Berkman T., Naftalovich R., Vatner D.E. (2020). Healthful Aging Mediated by Inhibition of Oxidative Stress. Ageing Res. Rev..

[42] Solhjoo S., Liu T., Sidor A., Lee D.I., O’Rourke B., Steenbergen C. (2023). Oxidative Stress in the Mitochondrial Matrix Underlies Ischemia/Reperfusion-Induced Mitochondrial Instability. J. Biol. Chem..

[43] Guo Y., Jia X., Cui Y., Song Y., Wang S., Geng Y., Li R., Gao W., Fu D. (2021). Sirt3-Mediated Mitophagy Regulates AGEs-Induced BMSCs Senescence and Senile Osteoporosis. Redox Biol..

[44] Zhang J., Wang H., Slotabec L., Cheng F., Tan Y., Li J. (2023). Alterations of SIRT1/SIRT3 Subcellular Distribution in Aging Undermine Cardiometabolic Homeostasis during Ischemia and Reperfusion. Aging Cell.

[45] Lin C.H., Li N.T., Cheng H.S., Yen M.L. (2018). Oxidative Stress Induces Imbalance of Adipogenic/Osteoblastic Lineage Commitment in Mesenchymal Stem Cells through Decreasing SIRT1 Functions. J. Cell. Mol. Med..

[46] Jiang Y., Luo W., Wang B., Wang X., Gong P., Xiong Y. (2020). Resveratrol Promotes Osteogenesis via Activating SIRT1/FoxO1 Pathway in Osteoporosis Mice. Life Sci..

[47] Gabay O., Zaal K.J., Sanchez C., Dvir-Ginzberg M., Gagarina V., Song Y., He X.H., McBurney M.W. (2013). Sirt1-Deficient Mice Exhibit an Altered Cartilage Phenotype. Jt. Bone Spine.

[48] Zainabadi K., Liu C.J., Caldwell A.L.M., Guarente L. (2017). SIRT1 Is a Positive Regulator of in Vivo Bone Mass and a Therapeutic Target for Osteoporosis. PLoS ONE.

[49] Chen Y., Tang W., Li H., Lv J., Chang L., Chen S. (2023). Composite Dietary Antioxidant Index Negatively Correlates with Osteoporosis among Middle-Aged and Older US Populations. Am. J. Transl. Res..

[50] Zhao F., Guo L., Wang X., Zhang Y. (2021). Correlation of Oxidative Stress-Related Biomarkers with Postmenopausal Osteoporosis: A Systematic Review and Meta-Analysis. Arch. Osteoporos..

[51] Chen T., Wang H., Jiang C., Lu Y. (2021). PKD1 Alleviates Oxidative Stress-Inhibited Osteogenesis of Rat Bone Marrow-Derived Mesenchymal Stem Cells through TAZ Activation. J. Cell. Biochem..

[52] Chen K., Qiu P., Yuan Y., Zheng L., He J., Wang C., Guo Q., Kenny J., Liu Q., Zhao J. (2019). Pseurotin A Inhibits Osteoclastogenesis and Prevents Ovariectomized-Induced Bone Loss by Suppressing Reactive Oxygen Species. Theranostics.

[53] Zou M.L., Chen Z.H., Teng Y.Y., Liu S.Y., Jia Y., Zhang K.W., Sun Z.L., Wu J.J., Yuan Z.D., Feng Y. (2021). The Smad Dependent TGF-β and BMP Signaling Pathway in Bone Remodeling and Therapies. Front. Mol. Biosci..

[54] Agidigbi T.S., Kim C. (2019). Reactive Oxygen Species in Osteoclast Differentiation and Possible Pharmaceutical Targets of ROS-Mediated Osteoclast Diseases. Int. J. Mol. Sci..

[55] Zhang X., Jiang Y., Mao J., Ren X., Ji Y., Mao Y., Chen Y., Sun X., Pan Y., Ma J. (2021). Hydroxytyrosol Prevents Periodontitis-Induced Bone Loss by Regulating Mitochondrial Function and Mitogen-Activated Protein Kinase Signaling of Bone Cells. Free. Radic. Biol. Med..

[56] Lee E., Moon J.Y., Ko J.Y., Park S.Y., Im G. (2023). Il GSTT1 as a Predictive Marker and Enhancer for Osteogenic Potential of Human Adipose-Derived Stromal/Stem Cells. J. Bone Miner. Res..

[57] Yang D.H., Chiang T.I., Chang I.C., Lin F.H., Wei C.C., Cheng Y.W. (2014). Increased Levels of Circulating Advanced Glycation End-Products in Menopausal Women with Osteoporosis. Int. J. Med. Sci..

[58] Tabara Y., Ikezoe T., Yamanaka M., Setoh K., Segawa H., Kawaguchi T., Kosugi S., Nakayama T., Ichihashi N., Tsuboyama T. (2019). Advanced Glycation End Product Accumulation Is Associated with Low Skeletal Muscle Mass, Weak Muscle Strength, and Reduced Bone Density: The Nagahama Study. J. Gerontol. A Biol. Sci. Med. Sci..

[59] Lu X., Min W., Shi Y., Tian L., Li P., Ma T., Zhang Y., Luo C. (2022). Exogenous Melatonin Alleviates Alkaline Stress by Removing Reactive Oxygen Species and Promoting Antioxidant Defence in Rice Seedlings. Front. Plant Sci..

[60] Yang K., Qiu X., Cao L., Qiu S. (2022). The Role of Melatonin in the Development of Postmenopausal Osteoporosis. Front. Pharmacol..

[61] Bantounou M., Plascevic J., Galley H.F. (2022). Melatonin and Related Compounds: Antioxidant and Anti-Inflammatory Actions. Antioxidants.

[62] Han H., Tian T., Huang G., Li D., Yang S. (2021). The LncRNA H19/MiR-541-3p/Wnt/β-Catenin Axis Plays a Vital Role in Melatonin-Mediated Osteogenic Differentiation of Bone Marrow Mesenchymal Stem Cells. Aging.

[63] Guan H., Kong N., Tian R., Cao R., Liu G., Li Y., Wei Q., Jiao M., Lei Y., Xing F. (2022). Melatonin Increases Bone Mass in Normal, Perimenopausal, and Postmenopausal Osteoporotic Rats via the Promotion of Osteogenesis. J. Transl. Med..

[64] Zhou Y., Wang C., Si J., Wang B., Zhang D., Ding D., Zhang J., Wang H. (2020). Melatonin Up-Regulates Bone Marrow Mesenchymal Stem Cells Osteogenic Action but Suppresses Their Mediated Osteoclastogenesis via MT2-Inactivated NF-ΚB Pathway. Br. J. Pharmacol..

[65] Liu H.D., Ren M.X., Li Y., Zhang R.T., Ma N.F., Li T.L., Jiang W.K., Zhou Z., Yao X.W., Liu Z.Y. (2022). Melatonin Alleviates Hydrogen Peroxide Induced Oxidative Damage in MC3T3-E1 Cells and Promotes Osteogenesis by Activating SIRT1. Free. Radic. Res..

[66] Chen G., Tang Q., Yu S., Xie Y., Sun J., Li S., Chen L. (2020). The Biological Function of BMAL1 in Skeleton Development and Disorders. Life Sci..

[67] Wang X., Liang T., Zhu Y., Qiu J., Qiu X., Lian C., Gao B., Peng Y., Liang A., Zhou H. (2019). Melatonin Prevents Bone Destruction in Mice with Retinoic Acid-Induced Osteoporosis. Mol. Med..

[68] Gürler E.B., Çilingir-Kaya Ö.T., Peker Eyüboglu I., Ercan F., Akkiprik M., Reiter R.J., Yegen B. (2019). Melatonin Supports Alendronate in Preserving Bone Matrix and Prevents Gastric Inflammation in Ovariectomized Rats. Cell Biochem. Funct..

[69] Gong W., Liu M., Zhang Q., Zhang Q., Wang Y., Zhao Q., Xiang L., Zheng C., Zhang Q., Qin L. (2022). Orcinol Glucoside Improves Senile Osteoporosis through Attenuating Oxidative Stress and Autophagy of Osteoclast via Activating Nrf2/Keap1 and MTOR Signaling Pathway. Oxid. Med. Cell. Longev..

[70] Zheng L., Zhuang Z., Li Y., Shi T., Fu K., Yan W., Zhang L., Wang P., Li L., Jiang Q. (2021). Bone Targeting Antioxidative Nano-Iron Oxide for Treating Postmenopausal Osteoporosis. Bioact. Mater..

[71] Tao Z.S., Li T.L., Wei S. (2022). Probucol Promotes Osteoblasts Differentiation and Prevents Osteoporosis Development through Reducing Oxidative Stress. Mol. Med..

[72] George K.S., Munoz J., Ormsbee L.T., Akhavan N.S., Foley E.M., Siebert S.C., Kim J.S., Hickner R.C., Arjmandi B.H. (2022). The Short-Term Effect of Prunes in Improving Bone in Men. Nutrients.

[73] Liu H., Zhao A., Huang Y., Hou A., Miao W., Hong L., Deng N., Fan Y. (2022). Efficacy and Mechanisms of Oleuropein in Postmenopausal Osteoporosis. Comput. Math. Methods Med..

[74] Jin C., Tan K., Yao Z., Lin B.H., Zhang D.P., Chen W.K., Mao S.M., Zhang W., Chen L., Lin Z. (2023). A Novel Anti-Osteoporosis Mechanism of VK2: Interfering with Ferroptosis via AMPK/SIRT1 Pathway in Type 2 Diabetic Osteoporosis. J. Agric. Food Chem..

[75] Roger L., Tomas F., Gire V. (2021). Mechanisms and Regulation of Cellular Senescence. Int. J. Mol. Sci..

[76] Calcinotto A., Kohli J., Zagato E., Pellegrini L., Demaria M., Alimonti A. (2019). Cellular Senescence: Aging, Cancer, and Injury. Physiol. Rev..

[77] Kumari R., Jat P. (2021). Mechanisms of Cellular Senescence: Cell Cycle Arrest and Senescence Associated Secretory Phenotype. Front. Cell Dev. Biol..

[78] Farr J.N., Khosla S. (2019). Cellular Senescence in Bone. Bone.

[79] Moerman E.J., Teng K., Lipschitz D.A., Lecka-Czernik B. (2004). Aging Activates Adipogenic and Suppresses Osteogenic Programs in Mesenchymal Marrow Stroma/Stem Cells: The Role of PPAR-Gamma2 Transcription Factor and TGF-Beta/BMP Signaling Pathways. Aging Cell.

[80] Abuna R.P.F., Stringhetta-Garcia C.T., Fiori L.P., Dornelles R.C.M., Rosa A.L., Beloti M.M. (2016). Aging Impairs Osteoblast Differentiation of Mesenchymal Stem Cells Grown on Titanium by Favoring Adipogenesis. J. Appl. Oral Sci..

[81] Kornicka K., Marycz K., Tomaszewski K.A., Marędziak M., Smieszek A. (2015). The Effect of Age on Osteogenic and Adipogenic Differentiation Potential of Human Adipose Derived Stromal Stem Cells (HASCs) and the Impact of Stress Factors in the Course of the Differentiation Process. Oxidative Med. Cell. Longev..

[82] Maredziak M., Marycz K., Tomaszewski K.A., Kornicka K., Henry B.M. (2016). The Influence of Aging on the Regenerative Potential of Human Adipose Derived Mesenchymal Stem Cells. Stem Cells Int..

[83] Yang Y.H.K., Ogando C.R., Wang See C., Chang T.Y., Barabino G.A. (2018). Changes in Phenotype and Differentiation Potential of Human Mesenchymal Stem Cells Aging in Vitro. Stem Cell Res. Ther..

[84] Kretlow J.D., Jin Y.Q., Liu W., Zhang W.J., Hong T.H., Zhou G., Baggett L.S., Mikos A.G., Cao Y. (2008). Donor Age and Cell Passage Affects Differentiation Potential of Murine Bone Marrow-Derived Stem Cells. BMC Cell Biol..

[85] Akter F., Tsuyama T., Yoshizawa T., Sobuz S.U., Yamagata K. (2021). SIRT7 Regulates Lipogenesis in Adipocytes through Deacetylation of PPARγ2. J. Diabetes Investig..

[86] Doolittle M.L., Monroe D.G., Farr J.N., Khosla S. (2021). The Role of Senolytics in Osteoporosis and Other Skeletal Pathologies. Mech. Ageing Dev..

[87] Zhang D., Yu K., Yang J., Xie S., Yang J., Tan L. (2020). Senolytic Controls Bone Marrow Mesenchymal Stem Cells Fate Improving Bone Formation. Am. J. Transl. Res..

[88] Kim H.N., Chang J., Shao L., Han L., Iyer S., Manolagas S.C., O’Brien C.A., Jilka R.L., Zhou D., Almeida M. (2017). DNA Damage and Senescence in Osteoprogenitors Expressing Osx1 May Cause Their Decrease with Age. Aging Cell.

[89] Kirkland J.L., Tchkonia T., Zhu Y., Niedernhofer L.J., Robbins P.D. (2017). The Clinical Potential of Senolytic Drugs. J. Am. Geriatr. Soc..

[90] Jing H., Liao L., An Y., Su X., Liu S., Shuai Y., Zhang X., Jin Y. (2016). Suppression of EZH2 Prevents the Shift of Osteoporotic MSC Fate to Adipocyte and Enhances Bone Formation During Osteoporosis. Mol. Ther..

[91] Reppe S., Lien T.G., Hsu Y.H., Gautvik V.T., Olstad O.K., Yu R., Bakke H.G., Lyle R., Kringen M.K., Glad I.K. (2017). Distinct DNA Methylation Profiles in Bone and Blood of Osteoporotic and Healthy Postmenopausal Women. Epigenetics.

[92] Fernandez-Rebollo E., Eipel M., Seefried L., Hoffmann P., Strathmann K., Jakob F., Wagner W. (2018). Primary Osteoporosis Is Not Reflected by Disease-Specific DNA Methylation or Accelerated Epigenetic Age in Blood. J. Bone Miner. Res..

[93] Xu F., Li W., Yang X., Na L., Chen L., Liu G. (2021). The Roles of Epigenetics Regulation in Bone Metabolism and Osteoporosis. Front. Cell Dev. Biol..

[94] Letarouilly J.G., Broux O., Clabaut A. (2019). New Insights into the Epigenetics of Osteoporosis. Genomics.

[95] Raje M.M., Ashma R. (2019). Epigenetic Regulation of BMP2 Gene in Osteoporosis: A DNA Methylation Study. Mol. Biol. Rep..

[96] Wakitani S., Yokoi D., Hidaka Y., Nishino K. (2017). The Differentially DNA-Methylated Region Responsible for Expression of Runt-Related Transcription Factor 2. J. Vet. Med. Sci..

[97] Wang R., Wang Y., Zhu L., Liu Y., Li W. (2020). Epigenetic Regulation in Mesenchymal Stem Cell Aging and Differentiation and Osteoporosis. Stem Cells Int..

[98] de Nigris F., Ruosi C., Colella G., Napoli C. (2021). Epigenetic Therapies of Osteoporosis. Bone.

[99] Hemming S., Cakouros D., Vandyke K., Davis M.J., Zannettino A.C.W., Gronthos S. (2016). Identification of Novel EZH2 Targets Regulating Osteogenic Differentiation in Mesenchymal Stem Cells. Stem Cells Dev..

[100] Zhu X.X., Yan Y.W., Chen D., Ai C.Z., Lu X., Xu S.S., Jiang S., Zhong G.S., Chen D.B., Jiang Y.Z. (2016). Long Non-Coding RNA HoxA-AS3 Interacts with EZH2 to Regulate Lineage Commitment of Mesenchymal Stem Cells. Oncotarget.

[101] Ren J., Huang D., Li R., Wang W., Zhou C. (2020). Control of Mesenchymal Stem Cell Biology by Histone Modifications. Cell Biosci..

[102] Huang M., Xu S., Liu L., Zhang M., Guo J., Yuan Y., Xu J., Chen X., Zou J. (2021). M6A Methylation Regulates Osteoblastic Differentiation and Bone Remodeling. Front. Cell Dev. Biol..

[103] Wu Y., Xie L., Wang M., Xiong Q., Guo Y., Liang Y., Li J., Sheng R., Deng P., Wang Y. (2018). Mettl3-Mediated M6A RNA Methylation Regulates the Fate of Bone Marrow Mesenchymal Stem Cells and Osteoporosis. Nat. Commun..

[104] Hagh M.F., Noruzinia M., Mortazavi Y., Soleimani M., Kaviani S., Abroun S., Fard A.D., Maymand M.M. (2015). Different Methylation Patterns of RUNX2, OSX, DLX5 and BSP in Osteoblastic Differentiation of Mesenchymal Stem Cells. Cell J..

[105] Cheishvili D., Parashar S., Mahmood N., Arakelian A., Kremer R., Goltzman D., Szyf M., Rabbani S.A. (2021). Identification of an Epigenetic Signature of Osteoporosis in Blood DNA of Postmenopausal Women. J. Bone Miner. Res..

[106] Visconti V.V., Cariati I., Fittipaldi S., Iundusi R., Gasbarra E., Tarantino U., Botta A. (2021). DNA Methylation Signatures of Bone Metabolism in Osteoporosis and Osteoarthritis Aging-Related Diseases: An Updated Review. Int. J. Mol. Sci..

[107] Li Y., Xie B., Jiang Z., Yuan B. (2019). Relationship between Osteoporosis and Osteoarthritis Based on DNA Methylation. Int. J. Clin. Exp. Pathol..

[108] Sabri S.A., Chavarria J.C., Ackert-Bicknell C., Swanson C., Burger E. (2023). Osteoporosis: An Update on Screening, Diagnosis, Evaluation, and Treatment. Orthopedics.

[109] Mäkitie R.E., Costantini A., Kämpe A., Alm J.J., Mäkitie O. (2019). New Insights Into Monogenic Causes of Osteoporosis. Front. Endocrinol..

[110] Mei Y., Zhang H., Zhang Z. (2022). Comparing Clinical and Genetic Characteristics of De Novo and Inherited COL1A1/COL1A2 Variants in a Large Chinese Cohort of Osteogenesis Imperfecta. Front. Endocrinol..

[111] Hu J., Lin X., Gao P., Zhang Q., Zhou B., Wang O., Jiang Y., Xia W., Xing X., Li M. (2023). Genotypic and Phenotypic Spectrum and Pathogenesis of WNT1 Variants in a Large Cohort of Patients with OI/Osteoporosis. J. Clin. Endocrinol. Metab..

[112] Zhu X., Bai W., Zheng H. (2021). Twelve Years of GWAS Discoveries for Osteoporosis and Related Traits: Advances, Challenges and Applications. Bone Res..

[113] Duncan E.L., Danoy P., Kemp J.P., Leo P.J., McCloskey E., Nicholson G.C., Eastell R., Prince R.L., Eisman J.A., Jones G. (2011). Genome-Wide Association Study Using Extreme Truncate Selection Identifies Novel Genes Affecting Bone Mineral Density and Fracture Risk. PLoS Genet.

[114] García-Ibarbia C., Pérez-Núñez M.I., Olmos J.M., Valero C., Pérez-Aguilar M.D., Hernández J.L., Zarrabeitia M.T., González-Macías J., Riancho J.A. (2013). Missense Polymorphisms of the WNT16 Gene Are Associated with Bone Mass, Hip Geometry and Fractures. Osteoporos. Int..

[115] Styrkarsdottir U., Halldorsson B.V., Gudbjartsson D.F., Tang N.L.S., Koh J.M., Xiao S.M., Kwok T.C.Y., Kim G.S., Chan J.C.N., Cherny S. (2010). European Bone Mineral Density Loci Are Also Associated with BMD in East-Asian Populations. PLoS ONE.

[116] Richards J., Rivadeneira F., Inouye M., Pastinen T., Soranzo N., Wilson S., Andrew T., Falchi M., Gwilliam R., Ahmadi K. (2008). Bone Mineral Density, Osteoporosis, and Osteoporotic Fractures: A Genome-Wide Association Study. Lancet.

[117] Zheng H.F., Forgetta V., Hsu Y.H., Estrada K., Rosello-Diez A., Leo P.J., Dahia C.L., Park-Min K.H., Tobias J.H., Kooperberg C. (2015). Whole-Genome Sequencing Identifies EN1 as a Determinant of Bone Density and Fracture. Nature.

[118] Guo Y., Yang T.L., Liu Y.Z., Shen H., Lei S.F., Yu N., Chen J., Xu T., Cheng Y., Tian Q. (2011). Mitochondria-Wide Association Study of Common Variants in Osteoporosis. Ann. Hum. Genet.

[119] Ma C., Yu R., Li J., Xiao E., Guo J., Wang X., Li G., Liu P. (2023). Cross-Sectional Study and Bioinformatics Analysis to Reveal the Correlations of Osteoporosis in Patients with Parkinson’s Disease. Exp. Gerontol..

[120] Han T., Zhang Y., Qi B., Chen M., Sun K., Qin X., Yang B., Yin H., Xu A., Wei X. (2023). Clinical Features and Shared Mechanisms of Chronic Gastritis and Osteoporosis. Sci. Rep..

[121] Xu D., Chen Y., Gao X., Xie W., Wang Y., Shen J., Yang G., Xie B. (2023). The Genetically Predicted Causal Relationship of Inflammatory Bowel Disease with Bone Mineral Density and Osteoporosis: Evidence from Two-Sample Mendelian Randomization. Front. Immunol..

[122] Jiang N., An J., Yang K., Liu J., Guan C., Ma C., Tang X. (2021). NLRP3 Inflammasome: A New Target for Prevention and Control of Osteoporosis?. Front. Endocrinol..

[123] Guaraná W.L., Lima C.A.D., Barbosa A.D., Crovella S., Sandrin-Garcia P. (2022). Can Polymorphisms in NLRP3 Inflammasome Complex Be Associated with Postmenopausal Osteoporosis Severity?. Genes.

[124] Iantomasi T., Romagnoli C., Palmini G., Donati S., Falsetti I., Miglietta F., Aurilia C., Marini F., Giusti F., Brandi M.L. (2023). Oxidative Stress and Inflammation in Osteoporosis: Molecular Mechanisms Involved and the Relationship with MicroRNAs. Int. J. Mol. Sci..

[125] Murakami T., Nakaminami Y., Takahata Y., Hata K., Nishimura R. (2022). Activation and Function of NLRP3 Inflammasome in Bone and Joint-Related Diseases. Int. J. Mol. Sci..

[126] Chen W., Tang P., Fan S., Jiang X. (2022). A Novel Inhibitor INF 39 Promotes Osteogenesis via Blocking the NLRP3/IL-1 β Axis. Biomed. Res. Int..

[127] Ono T., Hayashi M., Sasaki F., Nakashima T. (2020). RANKL Biology: Bone Metabolism, the Immune System, and Beyond. Inflamm. Regen..

[128] Huang F., Wong P., Li J., Lv Z., Xu L., Zhu G., He M., Luo Y. (2022). Osteoimmunology: The Correlation between Osteoclasts and the Th17/Treg Balance in Osteoporosis. J. Cell. Mol. Med..

[129] Lisco G., Triggiani D., Giagulli V.A., De Pergola G., Guastamacchia E., Piazzolla G., Jirillo E., Triggiani V. (2023). Endocrine, Metabolic, and Immune Pathogenesis of Postmenopausal Osteoporosis. Is There a Therapeutic Role in Natural Products?. Endocr. Metab. Immune Disord. Drug Targets.

[130] Lucas S., Omata Y., Hofmann J., Böttcher M., Iljazovic A., Sarter K., Albrecht O., Schulz O., Krishnacoumar B., Krönke G. (2018). Short-Chain Fatty Acids Regulate Systemic Bone Mass and Protect from Pathological Bone Loss. Nat. Commun..

[131] Russell R.G.G., Watts N.B., Ebetino F.H., Rogers M.J. (2008). Mechanisms of Action of Bisphosphonates: Similarities and Differences and Their Potential Influence on Clinical Efficacy. Osteoporos. Int..

[132] Dömötör Z.R., Vörhendi N., Hanák L., Hegyi P., Kiss S., Csiki E., Szakó L., Párniczky A., Erőss B. (2020). Oral Treatment with Bisphosphonates of Osteoporosis Does Not Increase the Risk of Severe Gastrointestinal Side Effects: A Meta-Analysis of Randomized Controlled Trials. Front. Endocrinol..

[133] Black D.M., Abrahamsen B., Bouxsein M.L., Einhorn T., Napoli N. (2019). Atypical Femur Fractures: Review of Epidemiology, Relationship to Bisphosphonates, Prevention, and Clinical Management. Endocr. Rev..

[134] Hervás A., Hermoso De Mendoza M., Bartolomé R., Forcén T. (2005). Osteoporosis Management in Primary Care. An. Sist. Sanit. Navar..

[135] Rinotas V., Liepouri F., Ouzouni M.-D., Chalkidi N., Papaneophytou C., Lampropoulou M., Vidali V.P., Kontopidis G., Couladouros E., Eliopoulos E. (2023). Structure-Based Discovery of Receptor Activator of Nuclear Factor-ΚB Ligand (RANKL)-Induced Osteoclastogenesis Inhibitors. Int. J. Mol. Sci..

[136] Zanchetta M.B., Boailchuk J., Massari F., Silveira F., Bogado C., Zanchetta J.R. (2018). Significant Bone Loss after Stopping Long-Term Denosumab Treatment: A Post FREEDOM Study. Osteoporos. Int..

[137] Ferrari S., Langdahl B. (2023). Mechanisms Underlying the Long-Term and Withdrawal Effects of Denosumab Therapy on Bone. Nat. Rev. Rheumatol..

[138] Martin T.J., Sims N.A., Seeman E. (2021). Physiological and Pharmacological Roles of PTH and PTHrP in Bone Using Their Shared Receptor, PTH1R. Endocr. Rev..

[139] Bandeira L., Michael Lewiecki E. (2022). Anabolic Therapy for Osteoporosis: Update on Efficacy and Safety. Arch. Endocrinol. Metab..

[140] Dito G., Lugaresi M., Degradi C., Guabello G., Longhi M., Corbetta S. (2023). Efficacy of Switching from Teriparatide to Zoledronic Acid or Denosumab on Bone Mineral Density and Biochemical Markers of Bone Turnover in Older Patients with Severe Osteoporosis: A Real-Life Study. Endocrine.

[141] van Dinther M., Zhang J., Weidauer S.E., Boschert V., Muth E.M., Knappik A., de Gorter D.J.J., van Kasteren P.B., Frisch C., Mueller T.D. (2013). Anti-Sclerostin Antibody Inhibits Internalization of Sclerostin and Sclerostin-Mediated Antagonism of Wnt/LRP6 Signaling. PLoS ONE.

[142] Aditya S., Rattan A. (2021). Sclerostin Inhibition: A Novel Target for the Treatment of Postmenopausal Osteoporosis. J. Midlife Health.

[143] Rodriguez B.S.Q., Correa R. (2023). Raloxifene. Xpharm Compr. Pharmacol. Ref..

[144] McLaughlin M.B., Awosika A.O., Jialal I. (2023). Calcitonin. StatPearls.

[145] Schroeder R.J., Staszkiewicz J., O’Quin C., Carroll B., Doan N., Patel S., Ahmadzadeh S., Kallurkar A., Viswanath O., Varrassi G. (2023). Oral Therapeutics Post Menopausal Osteoporosis. Cureus.

[146] Gao Z., Gao Z., Zhang H., Hou S., Zhou Y., Liu X. (2022). Targeting STING: From Antiviral Immunity to Treat Osteoporosis. Front. Immunol..

[147] Xu H., Wang W., Liu X., Huang W., Zhu C., Xu Y., Yang H., Bai J., Geng D. (2023). Targeting Strategies for Bone Diseases: Signaling Pathways and Clinical Studies. Signal Transduct. Target. Ther..

[148] Sindel D. (2023). Osteoporosis: Spotlight on Current Approaches to Pharmacological Treatment. Turk. J. Phys. Med. Rehabil..

[149] Eastell R., Rosen C.J., Black D.M., Cheung A.M., Murad M.H., Shoback D. (2019). Pharmacological Management of Osteoporosis in Postmenopausal Women: An Endocrine Society* Clinical Practice Guideline. J. Clin. Endocrinol. Metab..

[150] Kavanagh K.L., Guo K., Dunford J.E., Wu X., Knapp S., Ebetino F.H., Rogers M.J., Russell R.G.G., Oppermann U. (2006). The Molecular Mechanism of Nitrogen-Containing Bisphosphonates as Antiosteoporosis Drugs. Proc. Natl. Acad. Sci. USA.

[151] Ebetino F.H., Hogan A.M.L., Sun S., Tsoumpra M.K., Duan X., Triffitt J.T., Kwaasi A.A., Dunford J.E., Barnett B.L., Oppermann U. (2011). The Relationship between the Chemistry and Biological Activity of the Bisphosphonates. Bone.

[152] Drake M.T., Clarke B.L., Khosla S. (2008). Bisphosphonates: Mechanism of Action and Role in Clinical Practice. Mayo Clin. Proc. Mayo Clin..

[153] Everts-Graber J., Bonel H., Lehmann D., Gahl B., Häuselmann H.J., Studer U., Ziswiler H.R., Reichenbach S., Lehmann T. (2023). Comparison of Anti-Fracture Effectiveness of Zoledronate, Ibandronate and Alendronate versus Denosumab in a Registry-Based Cohort Study. Osteoporos. Int..

[154] Bone H.G., Wagman R.B., Brandi M.L., Brown J.P., Chapurlat R., Cummings S.R., Czerwiński E., Fahrleitner-Pammer A., Kendler D.L., Lippuner K. (2017). 10 Years of Denosumab Treatment in Postmenopausal Women with Osteoporosis: Results from the Phase 3 Randomised FREEDOM Trial and Open-Label Extension. Lancet Diabetes Endocrinol..

[155] Ding M., Cho E., Chen Z., Park S.W., Lee T.H. (2023). (S)-2-(Cyclobutylamino)-N-(3-(3,4-Dihydroisoquinolin-2(1H)-Yl)-2-Hydroxypropyl)Isonicotinamide Attenuates RANKL-Induced Osteoclast Differentiation by Inhibiting NF-ΚB Nuclear Translocation. Int. J. Mol. Sci..

[156] Mao Y., Xie X., Jiang T., Chao R., Wan T., Sun L., Sun G., Zhou Z., Xu W., Chen X. (2023). Xl019, a Novel JAK Inhibitor, Suppressed Osteoclasts Differentiation Induced by RANKL through MAPK Signaling Pathway. Biochem. Pharmacol..

[157] Ko Y.J., Sohn H.M., Jang Y., Park M., Kim B., Kim B., Park J., Hyun H., Jeong B., Hong C. (2021). A Novel Modified RANKL Variant Can Prevent Osteoporosis by Acting as a Vaccine and an Inhibitor. Clin. Transl. Med..

[158] Cupp M.E., Nayak S.K., Adem A.S., Thomsen W.J. (2013). Parathyroid Hormone (PTH) and PTH-Related Peptide Domains Contributing to Activation of Different PTH Receptor-Mediated Signaling Pathways. J. Pharmacol. Exp. Ther..

[159] Cipriani C., Pepe J., Silva B.C., Rubin M.R., Cusano N.E., McMahon D.J., Nieddu L., Angelozzi M., Biamonte F., Diacinti D. (2018). Comparative Effect of RhPTH(1-84) on Bone Mineral Density and Trabecular Bone Score in Hypoparathyroidism and Postmenopausal Osteoporosis. J. Bone Miner. Res..

[160] Nemec K., Schihada H., Kleinau G., Zabel U., Grushevskyi E.O., Scheerer P., Lohse M.J., Maiellaro I. (2022). Functional Modulation of PTH1R Activation and Signaling by RAMP2. Proc. Natl. Acad. Sci. USA.

[161] Vasiliadis E.S., Evangelopoulos D.S., Kaspiris A., Benetos I.S., Vlachos C., Pneumaticos S.G. (2022). The Role of Sclerostin in Bone Diseases. J. Clin. Med..

[162] Glass D.A., Bialek P., Ahn J.D., Starbuck M., Patel M.S., Clevers H., Taketo M.M., Long F., McMahon A.P., Lang R.A. (2005). Canonical Wnt Signaling in Differentiated Osteoblasts Controls Osteoclast Differentiation. Dev. Cell.

[163] Iolascon G., Liguori S., Paoletta M., Toro G., Moretti A. (2023). Anti-Sclerostin Antibodies: A New Frontier in Fragility Fractures Treatment. Ther. Adv. Musculoskelet. Dis..

[164] Korff C., Adaway M., Atkinson E.G., Horan D.J., Klunk A., Silva B.S., Bellido T., Plotkin L.I., Robling A.G., Bidwell J.P. (2023). Loss of Nmp4 Enhances Bone Gain from Sclerostin Antibody Administration. Bone.

[165] Lewiecki E.M., Dinavahi R.V., Lazaretti-Castro M., Ebeling P.R., Adachi J.D., Miyauchi A., Gielen E., Milmont C.E., Libanati C., Grauer A. (2019). One Year of Romosozumab Followed by Two Years of Denosumab Maintains Fracture Risk Reductions: Results of the FRAME Extension Study. J. Bone Miner. Res..

[166] Glorieux F.H., Devogelaer J.P., Durigova M., Goemaere S., Hemsley S., Jakob F., Junker U., Ruckle J., Seefried L., Winkle P.J. (2017). BPS804 Anti-Sclerostin Antibody in Adults With Moderate Osteogenesis Imperfecta: Results of a Randomized Phase 2a Trial. J. Bone Miner. Res..

[167] Su Y., Wang W., Liu F., Cai Y., Li N., Li H., Li G., Ma L. (2022). Blosozumab in the Treatment of Postmenopausal Women with Osteoporosis: A Systematic Review and Meta-Analysis. Ann. Palliat. Med..

[168] Jeong C., Ha J., Yoo J.-I., Lee Y.-K., Kim J.H., Ha Y.-C., Min Y.-K., Byun D.-W., Baek K.-H., Chung H.Y. (2023). Effects of Bazedoxifene/Vitamin D Combination Therapy on Serum Vitamin D Levels and Bone Turnover Markers in Postmenopausal Women with Osteopenia: A Randomized Controlled Trial. J. Bone Metab..

[169] D’Amelio P., Isaia G.C. (2013). The Use of Raloxifene in Osteoporosis Treatment. Expert Opin. Pharmacother..

[170] Ohta H., Uemura Y., Sone T., Tanaka S., Soen S., Mori S., Hagino H., Fukunaga M., Nakamura T., Orimo H. (2023). Effect of Bone Resorption Inhibitors on Serum Cholesterol Level and Fracture Risk in Osteoporosis: Randomized Comparative Study Between Minodronic Acid and Raloxifene. Calcif. Tissue Int..

[171] Muñoz-Torres M., Alonso G., Mezquita Raya P. (2004). Calcitonin Therapy in Osteoporosis. Treat. Endocrinol..

[172] Balka K.R., De Nardo D. (2021). Molecular and Spatial Mechanisms Governing STING Signalling. FEBS J..

[173] Alto L.T., Terman J.R. (2017). Semaphorins and Their Signaling Mechanisms. Methods Mol. Biol..

[174] Negishi-Koga T., Shinohara M., Komatsu N., Bito H., Kodama T., Friedel R.H., Takayanagi H. (2011). Suppression of Bone Formation by Osteoclastic Expression of Semaphorin 4D. Nat. Med..

[175] Ishii T., Ruiz-Torruella M., Yamamoto K., Yamaguchi T., Heidari A., Pierrelus R., Leon E., Shindo S., Rawas-Qalaji M., Pastore M.R. (2022). Locally Secreted Semaphorin 4D Is Engaged in Both Pathogenic Bone Resorption and Retarded Bone Regeneration in a Ligature-Induced Mouse Model of Periodontitis. Int. J. Mol. Sci..

[176] Hayashi M., Nakashima T., Taniguchi M., Kodama T., Kumanogoh A., Takayanagi H. (2012). Osteoprotection by Semaphorin 3A. Nature.

[177] Geng Q., Gao H., Yang R., Guo K., Miao D. (2019). Pyrroloquinoline Quinone Prevents Estrogen Deficiency-Induced Osteoporosis by Inhibiting Oxidative Stress and Osteocyte Senescence. Int. J. Biol. Sci..

[178] Huang Y., Chen N., Miao D. (2017). Effect and Mechanism of Pyrroloquinoline Quinone on Anti-Osteoporosis in Bmi-1 Knockout Mice-Anti-Oxidant Effect of Pyrroloquinoline Quinone. Am. J. Transl. Res..

[179] Wu X., Li J., Zhang H., Wang H., Yin G., Miao D. (2017). Pyrroloquinoline Quinone Prevents Testosterone Deficiency-Induced Osteoporosis by Stimulating Osteoblastic Bone Formation and Inhibiting Osteoclastic Bone Resorption. Am. J. Transl. Res..

[180] Deng Y., Wei W., Tang P. (2022). Applications of Calcium-Based Nanomaterials in Osteoporosis Treatment. ACS Biomater. Sci. Eng..

[181] Meng F., Yu Y., Tian Y., Deng M., Zheng K., Guo X., Zeng B., Li J., Qian A., Yin C. (2023). A Potential Therapeutic Drug for Osteoporosis: Prospect for Osteogenic LncRNAs. Front. Endocrinol..

[182] Wu Z., Zhu J., Wen Y., Lei P., Xie J., Shi H., Wu R., Lou X., Hu Y. (2023). Hmga1-Overexpressing Lentivirus Protects against Osteoporosis by Activating the Wnt/β-Catenin Pathway in the Osteogenic Differentiation of BMSCs. FASEB J..

[183] Oh W.T., Yang Y.S., Xie J., Ma H., Kim J.M., Park K.H., Oh D.S., Park-Min K.H., Greenblatt M.B., Gao G. (2023). WNT-Modulating Gene Silencers as a Gene Therapy for Osteoporosis, Bone Fracture, and Critical-Sized Bone Defects. Mol. Ther..

[184] Liang B., Burley G., Lin S., Shi Y.C. (2022). Osteoporosis Pathogenesis and Treatment: Existing and Emerging Avenues. Cell. Mol. Biol. Lett..

[185] Li J., Zhou Z., Wen J., Jiang F., Xia Y. (2020). Human Amniotic Mesenchymal Stem Cells Promote Endogenous Bone Regeneration. Front. Endocrinol..

[186] Kushioka J., Chow S.K.H., Toya M., Tsubosaka M., Shen H., Gao Q., Li X., Zhang N., Goodman S.B. (2023). Bone Regeneration in Inflammation with Aging and Cell-Based Immunomodulatory Therapy. Inflamm. Regen..

[187] Marozik P., Alekna V., Rudenko E., Tamulaitiene M., Rudenka A., Mastaviciute A., Samokhovec V., Cernovas A., Kobets K., Mosse I. (2019). Bone Metabolism Genes Variation and Response to Bisphosphonate Treatment in Women with Postmenopausal Osteoporosis. PLoS ONE.

[188] Villagómez Vega A., Gámez Nava J.I., Ruiz González F., Pérez Romero M., Trujillo Rangel W.Á., Nuño Arana I. (2023). Influence of the Osteogenomic Profile in Response to Alendronate Therapy in Postmenopausal Women with Osteoporosis: A Retrospective Cohort Study. Genes.

[189] Marcucci G., Domazetovic V., Nediani C., Ruzzolini J., Favre C., Brandi M.L. (2023). Oxidative Stress and Natural Antioxidants in Osteoporosis: Novel Preventive and Therapeutic Approaches. Antioxidants.

[190] Wang J., Shu B., Tang D.Z., Li C.G., Xie X.W., Jiang L.J., Jiang X.B., Chen B.L., Lin X.C., Wei X. (2023). The Prevalence of Osteoporosis in China, a Community Based Cohort Study of Osteoporosis. Front. Public Health.

[191] Ahire J.J., Kumar V., Rohilla A. (2023). Understanding Osteoporosis: Human Bone Density, Genetic Mechanisms, Gut Microbiota, and Future Prospects. Probiotics Antimicrob Proteins.

[192] Anupama D.S., Noronha J.A., Acharya K.K.V., Prabhu M., Ravishankar N., Nayak B.S. (2023). Effect of Lifestyle Modification Intervention Programme on Bone Mineral Density among Postmenopausal Women with Osteoporosis. Sultan Qaboos Univ. Med. J..

[193] Nield L., Bowles S.D. (2023). Assessment, Treatment and Prevention of Vitamin D Deficiency. Nurs. Stand..

[194] Singh A., Varma A.R. (2023). Whole-Body Vibration Therapy as a Modality for Treatment of Senile and Postmenopausal Osteoporosis: A Review Article. Cureus.

[195] Awuti K., Wang X., Sha L., Leng X. (2022). Exploring the Regulatory Mechanism of Osteoporosis Based on Intestinal Flora: A Review. Medicine.

[196] Zhang Y.W., Cao M.M., Li Y.J., Zhang R.L., Wu M.T., Yu Q., Rui Y.F. (2022). Fecal Microbiota Transplantation as a Promising Treatment Option for Osteoporosis. J. Bone Miner. Metab..

